# Maternal and Perinatal Outcomes of Pregnant Patients with Coronavirus Disease 2019: Data from a University Hospital Setting in Tirana, Albania, May 2020 to November 2021

**DOI:** 10.1155/2023/4032010

**Published:** 2023-06-14

**Authors:** Enkeleda Prifti, Najada Como, Enxhi Vrapi, Alketa Hoxha Qosja, Evelina Kreko, Nevila Kryemadhi, Elizana Petrela, Irsida Mehmeti, Genci Hyska

**Affiliations:** ^1^University of Medicine Tirana, Albania; ^2^Service of Obstetrics and Gynecology, University Hospital of Obstetrics and Gynecology ‘Koço Gliozheni', Tirana, Albania; ^3^Service of Infectious Disease, University Hospital ‘Nënë Tereza', Tirana, Albania; ^4^Faculty of Pharmacy, Catholic University Our Lady of Good Counsel, Tirana, Albania; ^5^Service of Anesthesiology and Reanimation, University Hospital of Obstetrics and Gynecology ‘Koço Gliozheni', Tirana, Albania

## Abstract

Scientific evidence suggests an increased risk of maternal and obstetric complications in pregnant patients infected with severe acute respiratory syndrome coronavirus 2 (SARS-CoV-2). This study is aimed at evaluating perinatal and maternal outcomes among patients with coronavirus disease 2019 (COVID-19) in a university hospital setting. This was a prospective cohort study of 177 pregnant women with confirmed SARS-CoV-2 infection at a tertiary hospital between May 2020 and November 2021. Both symptomatic and asymptomatic women with a positive reverse transcription-polymerase chain reaction test result at any time during pregnancy were included in this study. For the purpose of this study, we classified COVID-19 cases into two groups: mild and severe cases. The two groups were then compared to predict how the clinical presentation of COVID-19 affected adverse maternal and perinatal outcomes. Gestational age ≥ 20 weeks at the time of infection was significantly associated with the occurrence of severe forms of the disease (relative risk (RR) 3.98, *p* = 0.01). Cesarean section was the preferred mode of delivery, with 95 women (62.1%) undergoing surgery. A total of 149 neonates were delivered to women who had confirmed SARS-CoV-2 infection at any time during the course of pregnancy of which thirty-five (23.5%) were admitted to the neonatal intensive care unit (NICU). Severe forms of COVID-19 increased the risk of premature delivery (RR 6.69, *p* < 0.001), emergency cesarean delivery (RR 9.4, *p* < 0.001), intensive care hospitalization (RR 51, *p* < 0.001), and maternal death (RR 12.3, *p* = 0.02). However, severe forms of SARS-CoV-2 infection are not directly responsible for low birth weight or the need for neonatal resuscitation. Our findings suggest that pregnant women presenting with severe COVID-19 disease are at an increased risk of adverse maternal and perinatal outcomes, such as premature delivery, cesarean section, admission to the ICU, and maternal death. Infection after the 20^th^ week of gestation increases the risk of developing severe forms of the disease.

## 1. Introduction

Even after two years from the first case identified in Wuhan, China, the COVID-19 pandemic is still considered a public health emergency. Since the first reported COVID-19 case on March 9, 2020, in Albania, the virus has rapidly spread across the country reaching 292,456 confirmed cases and 3,517 deaths nationwide until July 14, 2022 [1]. The changes to the healthcare system, due to pandemic, presented a challenge for hospital interactions, especially for cases with severe forms of coronavirus disease 2019.

Especially, the management of infected pregnant women during the COVID-19 pandemic presented a challenge for obstetricians because of the unexpected adverse outcomes. Although pregnant women with COVID-19 might need hospitalization and admission to an intensive care unit (ICU), they are less likely than nonpregnant women to report symptoms. Therefore, many researchers have focused on possible obstetric and neonatal complications in pregnant women with coronavirus disease 2019 (COVID-19). Data from multicenter studies demonstrate that pregnant women with severe forms of COVID-19 during the late second and early third trimesters are at increased risk of adverse obstetric and neonatal outcomes [2].

This study is aimed at evaluating maternal and perinatal outcomes and risk factors of severe acute respiratory syndrome coronavirus 2 (SARS-CoV-2) infection in patients admitted to our university hospital of obstetrics and gynecology in Tirana, Albania.

Primarily, the relationship between maternal infection with SARS-CoV-2 and maternal/perinatal outcomes was investigated. Secondarily, the implication of several possible risk factors in the severity of infection was studied.

This is the first and the largest study of COVID-19 impact on pregnancy outcomes in Albania, a middle-income country, where epidemiological reports are scant.

## 2. Materials and Methods

This was a prospective cohort study of diagnosed cases of SARS-CoV-2 infection at the University Hospital of Obstetrics and Gynecology “Koço Gliozheni,” a tertiary hospital in Tirana, Albania, from May 2020 to November 2021. The diagnosed cases were thereafter referred to two COVID-19 designated hospitals to receive specialized care: the Infectious Disease Unit at the University Hospital “Nënë Tereza” and the University Hospital “Shefqet Ndroqi.”

Both symptomatic and asymptomatic women who had a positive reverse transcription-polymerase chain reaction test result at the state institutions or privately, in a licensed laboratory, at any time during pregnancy were included in this study. On the other hand, the patients with negative COVID-19 molecular tests were excluded. The inability to prospectively follow up cases for up to 40 days postpartum also served as an exclusion criterion.

For the purpose of this study, we classified COVID-19 cases into two groups: mild and severe cases.

Both asymptomatic and cases presenting with either fever, cough, fatigue, loss of taste or smell and shortness of breath, or pneumonia with O_2_saturation > 95%, or a combination of the above, were classified as mild.

We defined severe COVID-19 cases with the presence of severe dyspnea, respiratory rate of >30/min, O_2_saturation < 95%, and need for high-flow oxygen therapy or ICU admission.

The study evaluated perinatal (preeclampsia/eclampsia, prematurity, emergency cesarean section, and intrauterine fetal demise), maternal (intensive care unit admission, mechanical ventilation, and death), and neonatal (birth weight < 2500 g, neonatal intensive care admission, and death) outcomes.

Baseline characteristics such as age, BMI, parity, history of preexisting medical conditions, and gestational age at diagnosis were also noted.

Patients who provided informed consent were included in the study.

Staff members of the university hospital contributed by gathering patient data, under the guidance of the authors of the study.

Personal data were coded to mask patients' identities and stored in a secured database. This study was approved by the department of obstetrics and gynecology at our institution.

Statistical analysis was performed with STATA/SE version 15 software package and XLMiner extension pack for Excel 2016, developed by Analytic Solver Data Mining. Collinearity between independent variables was tested to avoid the risk of overrepresentation. The chi-square test was performed for categorical variables, whereas Mann-Whitney *U* tests were performed for ordinal and continuous variables. More specifically, logistic regression was used to evaluate the contribution of gestational age, age, BMI, and preexisting medical conditions to maternal outcomes.

Logistic regression analysis was also used to evaluate the contribution of severe COVID-19 in adverse maternal and neonatal outcomes. With a 95% confidence interval, the odds ratio was estimated to determine the constant effect of the above-mentioned characteristics on the likelihood of maternal outcomes. Statistical significance was defined as *p* < 0.05.

## 3. Results

Among the 177 patients who met the inclusion criteria, 162 (92%) were diagnosed during pregnancy and 15 (8%) during puerperium. Seventy-four percent (131/177) of the women had mild disease, whereas 26% (45/177) presented with severe COVID-19. The mean maternal age was 29.88 ± 5.46 years. Of the women, 37.95% were obese and only 6.2% had preexisting medical conditions.

Neither age nor BMI affected the clinical course of SARS-CoV-2 infection in the women in our cohort. The mean gestational age at diagnosis was calculated as 30.2 ± 9.77 weeks for mild and 30.86 ± 6.74 weeks for severe cases, with no significant difference between the groups. However, the risk of severe disease was 4-fold higher (adjusted odds ratio [aOR] 3.82, confidence interval [CI] 1.27–11.45, *p* = 0.01) for women diagnosed at ≥20 weeks of gestation, as shown in Table 1.

As for obstetric outcomes, summarized in Table 2, prematurity was statistically higher among women with severe disease (aOR 6.6, CI 2.7–16.01, *p* < 0.001). The mean gestational age at delivery was 37.33 ± 3.87 and 35.57 ± 3.64 weeks for the mild and the severe groups, respectively (*p* < 0.001). Intrauterine fetal demise and miscarriage risk were not statistically different between the two groups.

Cesarean section was the preferred mode of delivery, with 95 women (62.1%) undergoing surgery. In 31 cases (32.6%), the procedure was performed in an emergency setting. The severity of the disease significantly increased the risk of emergency cesarean section (aOR 9.39, CI 3.52–24.9, *p* < 0.001).

Fourteen women (7.9%) were admitted to an intensive care unit in need of specialized care due to disease progression and resistant hypoxia. Mechanical ventilation was required in six cases (3.4%). Despite this, there were five registered cases of maternal death: four women with severe disease and one patient who initially presented with mild COVID-19, and clinical deterioration was fulminant. The most common complication was interstitial pneumonia (27.1%) followed by respiratory failure (6.2%).

Detailed maternal and neonatal results are depicted in Tables 3 and 4.

A total of 149 neonates were delivered to women who had confirmed SARS-CoV-2 infection at any time during the course of pregnancy. The mean birth weight was 3105.7 ± 700 and 2673 ± 838 g for mild and severe cases, respectively (*p* < 0.001). Thirty-five neonates (23.5%) were admitted to the neonatal intensive care unit (NICU); however, no significant difference was noted between the two groups in terms of NICU admission.

The logistic regression analysis, reflected in Tables 5 and 6, respectively, concluded that severe disease significantly increased the odds of unfavorable maternal outcomes, such as intensive care unit (ICU) admission (aOR 57.36, *p* < 0.01) and maternal death (aOR 13.75, *p* < 0.01) but was not directly culpable for adverse neonatal outcomes (aOR 1.65, *p* = 0.46).

## 4. Discussion

In this cohort of 177 pregnant women with confirmed COVID-19, 46 (25.9%) had severe disease. The rate of severe disease is higher to what has been reported in other observational studies and in recent meta-analysis that estimates it to be between 9% and 13% (95% CI 6-21%) [3, 4].

In this study, severe COVID-19 was strongly associated with prematurity, increased cesarean sections, and maternal ICU admissions. These correlations appear similar to what has been previously described in a systematic review and meta-analysis [5]. Wei et al. also describe increased risk of preeclampsia in patients with severe disease, but this was not observed in our results.

Advanced gestational age (>20 weeks) was identified as a risk factor for severity of infection. Factors such as age > 35 years, preexisting disease, and BMI > 30 that have been acknowledged as risk factors by Allotey et al. were not found to statistically increase disease severity in our cohort.

The trend of infections in pregnant patients in Albania coincided with that of new cases in the population, where 69.5% of the patients were diagnosed over two periods with increasing cases: January-March 2021 and August-November 2021. Both periods coincided with the turnover of beta and delta variants [6].

Initially, limited testing was offered only to patients with severe symptomatology, and their contacts underestimated the real number of SARS-CoV-2 infections in pregnant women. A survey reported that the prevalence of infections in women presenting to obstetric hospitals varied between 3% and 20% [7, 8]. As previously reported, the lack of extensive testing in the population limited the comparison of results from obstetric patients with those of the general population [7, 8].

Limited testing may have left asymptomatic pregnant women unidentified. According to a study in the USA, asymptomatic cases are 15 times more common in pregnant women [4, 9]. A systematic review of the literature reported that 73% of pregnant women were asymptomatic [4]. In our study, only 39 patients (22%) were asymptomatic. The discrepancy in these results indicates obstacles in the provision of tests, which is a limitation of this study. In addition to the low percentage of asymptomatic cases, a small number of moderate cases were noted in our study.

Differentiating between moderate and severe forms, though theoretically well defined, is difficult to verify clinically where diagnostic tools for pneumonia (computed tomography scan and radiography) are not routinely offered to pregnant women. Another possible limitation of the study, also recognized in the literature, was the inability to distinguish between spontaneous and iatrogenic premature deliveries.

Factors such as age (mean 29.88 ± 5.46 years), BMI over 30 (37.85% of cases), a positive medical history of chronic diseases (6.2%), and parity did not significantly increase the likelihood of developing severe forms of the disease, despite literature describing these as possible risk factors [10–13].

Gestational age at the time of infection affected the severity of SARS-CoV-2 infection. A cohort study conducted at four European university hospitals reported worsening symptomatology and an increase in adverse obstetric outcomes in cases diagnosed after the 20th gestational week [1]. We used this threshold to calculate the risk in our cohort, where after corrections for possible confounding factors, a late diagnosis was associated with a 4-fold increased risk of occurrence of severe forms of the disease (OR 3.98, CI 1.26–11.99, *p* = 0.01).

The literature reports that SARS-CoV-2 infection during pregnancy, especially in those presenting with severe disease, increases the risk of perinatal complications such as preeclampsia, prematurity, and intrauterine fetal demise [14–18]. In our cohort, no statistically significant relationship was found between severe forms of infection and preeclampsia or fetal demise. However, prematurity was 26.8%, and its incidence was closely related to severity of the disease even after adjustment for possible confounding factors (OR 6.69, CI 2.79–16.01, *p* < 0.001).

The increased risk of adverse obstetric outcomes is directly associated with cesarean section [14, 19]. Cesarean sections should not be performed for all patients with SARS-CoV-2 as it does not serve as a protective factor against the transmission of infection [20, 21]. In addition, birth should be accelerated only in the case of severe disease to help improve maternal respiratory function.

The percentage of births with cesarean section was 58.6% in our study, which was up to 40% higher than the annual incidences at our institution. Severity of COVID-19, particularly compromised maternal respiratory function, was associated with a 6-fold increased risk of undergoing cesarean section (*p* = 0.04).

A body of evidence suggests that SARS-CoV-2 infection during pregnancy increases the risk of intensive care admission, the need for mechanical ventilation, and maternal death [4, 14, 22–24]. In this study, pregnant women presenting with severe COVID-19 had a higher risk of ICU admission (aOR 57.36, *p* < 0.01) and death (aOR 13.75, *p* < 0.01).

In terms of neonatal outcomes, although a statistical difference in birth weight was noted between patients with mild and severe forms of the disease, after a detailed analysis of confounding factors, it was found that severe forms of COVID-19 were not directly responsible for low birth weight or the need for neonatal intensive care. Neonatal morbidity was mainly associated with premature birth [4, 25, 26].

This study has various limitations. The results were gathered from only one tertiary hospital and its two referral centers. Though it helped improve data quality and consistency, this limits the generalizability of the results. The results reflect only on patients admitted to a tertiary hospital, thus underestimating the real burden of asymptomatic/mild cases who did not require inpatient care. Another limitation is the small sample size, which is conditioned by the population of Albania, and the insufficient of testing and identification of cases, especially at the beginning of the pandemic.

## 5. Conclusions

Our findings suggest that pregnant women presenting with severe COVID-19 disease are at an increased risk of adverse maternal and perinatal outcomes, such as premature delivery, cesarean section, admission to the ICU, and maternal death. Babies delivered to mothers with severe disease are more prone for adverse neonatal outcomes. Infection after the 20^th^ week of gestation increases the risk of developing severe forms of the disease.

Women should therefore be informed about the increased risk brought upon by severe forms of COVID-19 infection. Despite advanced gestation being identified as a possible contributor to severe disease, predictability of severity remains inaccurate. Therefore, COVID-19 prevention remains key in diminishing adverse perinatal outcomes.

## Figures and Tables

**Table 1:** Baseline characteristics of women with confirmed SARS-CoV-2 infection.

	Total (*n* = 177) (%)	COVID-19 cases		*p* ^∗^
		Mild (*n* = 131) (%)	Severe (*n* = 46) (%)	
Mean maternal age	29.88 ± 5.46	29.7 ± 5.23	30.34 ± 6.08	0.497
BMI (mean)	29.16 ± 3.98	29.11 ± 3.9	29.2 ± 4.1	0.29
BMI				
≥30	67 (37.85%)	51 (91.04)	16 (8.96)	0.754
≥40	6 (3.38%)	5 (83.3%)	1 (16.7%)	0.6
Preexisting medical conditions				
No	166 (93.8%)	120 (72.3%)	45 (27.7%)	0.18
Yes	11 (6.2%)	10 (90.9%)	1 (9.1%)	
Parity				
Primiparous	102 (57.6%)	79 (77.4%)	23 (22.6%)	0.22
Pluriparous	75 (42.4%)	52 (69.3%)	23 (30.7%)	
Gestational age at diagnosis				
Mean	30.41 ± 9.04	30.2 ± 9.77	30.86 ± 6.74	0.44
≥20 weeks	138 (77.96%)	96 (69.5%)	42 (30.5%)	0.01
≥24 weeks	129 (72.88)	91 (70.5%)	38 (29.5%)	0.08
≥32 weeks	95 (53.67%)	68 (71.6%)	27 (28.4%)	0.42
Puerperium	15 (8.47%)	13 (86.6%)	2	0.25

^∗^Statistically significant values at *p* < 0.05. Chi-square test was used for cardinal variables; the Mann-Whitney *U* test was used for continuous variables. COVID-19: coronavirus disease 2019; SARS-CoV-2: severe acute respiratory syndrome coronavirus 2; BMI: body mass index.

**Table 2:** Obstetric characteristics and outcomes of women with confirmed SARS-CoV-2 infection.

	Total (*n* = 162) (%)^a^	COVID-19 cases		*p* ^∗^
		Mild (*n* = 118) (%)	Severe (*n* = 44) (%)	
Delivery				
Term	112 (73.2%)	92 (82.1%)	20 (19.9%)	<0.001
Premature	41 (26.8%)	19 (46.4%)	22 (53.7%)	
Gestational age at delivery				
Mean	36.84 ± 3.88	37.33 ± 3.87	35.57 ± 3.64	<0.001
20–24 weeks	3 (1.9%)	3 (100%)	0	
24–32 weeks	13 (8.5%)	7 (53.5%)	6 (46.5%)	
32–37 weeks	25 (16.3%)	9 (36%)	16 (64%)	
≥37 weeks	112 (73.3%)	92 (82.1%)	20 (17.9%)	
Intrauterine fetal demise	8 (5.2%)	6 (75%)	2 (25%)	0.8
Miscarriage	9 (5.5%)	7 (77.8%)	2 (22.2%)	0.9
Mode of delivery				
Vaginal	58 (37.9%)	50 (86.2%)	8 (13.8%)	0.004
C-section	95 (62.1%)	61 (64.2%)	34 (35.8%)	
Cesarean section				
Elective	64 (67.4%)	50 (78.1%)	14 (21.9%)	<0.001
Emergency	31 (32.6%)	11 (35.5%)	20 (64.5%)	
Maternal indication	17 (17.9%)	0	17 (100%)	0.001
Obstetric conditions				
Preeclampsia	6 (3.9%)	4 (66.7%)	2 (33.3%)	0.7
IH cholestasis	1 (0.65%)	1 (100%)	0	0.9

^∗^Statistically significant values at *p* < 0.05. Chi-square test was used for cardinal variables; the Mann-Whitney *U* test was used for continuous variables. ^a^Based on total births, excluding puerperal women. IH: intrahepatic; COVID-19: coronavirus disease 2019; SARS-CoV-2: severe acute respiratory syndrome coronavirus 2; C-section: cesarean section.

**Table 3:** Maternal outcomes in women with confirmed SARS-CoV-2 infection.

	Total (*n* = 177)	COVID-19 cases		*p* ^∗^
		Mild (*n* = 131)	Severe (*n* = 46)	
ICU	14 (7.9%)	1 (7.1%)	13 (92.9%)	<0.001
High-flow O_2_ therapy	5 (2.8%)	1 (20%)	4 (80%)	0.02
CPAP	8 (4.5%)	0	8 (100%)	0.005
Mechanical ventilation	6 (3.4%)	1 (16.7%)	5 (83.3%)	0.01
Maternal death	5 (2.8%)	1 (20%)	4 (80%)	0.02
Complications				
Interstitial pneumonia	48 (27.1%)	5 (10.4%)	43 (89.6%)	<0.001
Respiratory failure	11 (6.2%)	0	11(100%)	0.002
Thromboembolism	3 (1.7%)	0	3 (100%)	0.04
Cardiac failure	2 (1.1%)	0	2 (100%)	0.08
Multiorgan failure	1 (0.6%)	0	1 (100%)	0.1

^∗^Statistically significant values at *p* < 0.05. Chi-square test was used for cardinal variables. ICU: intensive care unit; CPAP: continuous positive airway pressure; COVID-19: coronavirus disease 2019; SARS-CoV-2: severe acute respiratory syndrome coronavirus 2.

**Table 4:** Neonatal outcomes of infants delivered to women with confirmed SARS-CoV-2 infection.

	Total (*n* = 149^a^)	COVID-19		*p* ^∗^
		Mild (*n* = 107) (71.8%)	Severe (*n* = 42) (28.2%)	
Birth weight ≤ 2500 g	23 (15.4%)	12 (52.8%)	11 (47.2%)	0.02
Mean birth weight	2983.9 ± 764	3105.7 ± 700	2673 ± 838	<0.001
Apgar 5′ < 7	5 (3.4%)	1 (20%)	4 (80%)	0.03
NICU	35 (23.5%)	19 (54.3%)	16 (45.7%)	0.009
Neonatal death	4 (2.7%)	3 (75%)	1 (25%)	0.8

^∗^Statistically significant values at *p* < 0.05. Chi-square test was used for cardinal variables; the Mann-Whitney *U* test was used for continuous variables. ^a^Based on 149 children born to 145 women (intrauterine fetal demise excluded). NICU: neonatal intensive care unit; COVID-19: coronavirus disease 2019; SARS-CoV-2: severe acute respiratory syndrome coronavirus 2.

**Table 5:** Logistic regression analysis of maternal outcomes.

Maternal outcomes						
ICU admission				Maternal death		
	aOR	CI	*p*	aOR	CI	*p* ^∗^
Age	0.94	0.84-1.05	0.32	0.81	0.74-0.89	<0.01
BMI	2.78	0.73-10.57	0.13	8.5	1.01-71.6	0.04
Preexisting medical conditions	<0.01	0-infinity	0.99	<0.01	0-infinity	0.98
Obstetrical complications	1.36	0.07-25.13	0.83	<0.01	0-infinity	0.99
Severe COVID-19	57.36	6.81-478.9	<0.01	13.75	1.78-106.1	0.01

^∗^Statistically significant values at *p* < 0.05. aOR adjusted for age, BMI, preexisting medical conditions, and obstetrical complications. ICU: intensive care unit; aOR: adjusted odds ratio; CI: confidence interval; BMI: body mass index; COVID-19: coronavirus disease 2019.

**(a):** 

Neonatal outcomes			
Prematurity			
	aOR	CI	*p* ^∗^
Severe COVID-19	6.69	2.79-16.01	<0.001
Age	1.04	0.96-1.14	0.2
BMI	0.88	0.35-2.16	0.7
Preexisting medical conditions	0.94	0.13-6.44	0.9
Parity	0.61	0.61-4.3	0.3
Obstetric complications	34.9	3.86-315.9	<0.001

**(b):** 

	aOR	CI	*p* ^∗^
Birth weight < 2500 g			
Severe COVID-19	1.65	0.42-6.47	0.46
Age	1.31	1.14-1.49	<0.01
Emergency CS	3.1	0.81-12	0.09
Parity	0.2	0.04-0.82	0.02
Gestational age	0.75	0.66-0.84	<0.01

NICU			
Severe COVID-19	1.18	0.35-3.89	0.73
Age	0.98	0.89-1.07	0.68
Emergency CS	5.02	1.49-16.8	0.008
Weight < 2500 g	27.6	6.49-117	0.16
Obstetric complications	3.62	0.6-21.8	0.16

^∗^Statistically significant values at *p* < 0.05. aOR adjusted for age, BMI, parity, preexisting medical conditions, and gestational age. aOR: adjusted odds ratio; CI: confidence interval; BMI: body mass index; NICU: neonatal intensive care unit; CS: cesarean section; COVID-19: coronavirus disease 2019.

## Data Availability

All relevant data are within the paper and its supporting information files.
